# The integrated analysis of gut microbiota and metabolome revealed steroid hormone biosynthesis is a critical pathway in liver regeneration after 2/3 partial hepatectomy

**DOI:** 10.3389/fphar.2024.1407401

**Published:** 2024-08-12

**Authors:** Runbin Sun, Fei Fei, Dandan Jin, Haoyi Yang, Zhi Xu, Bei Cao, Juan Li

**Affiliations:** ^1^ Phase I Clinical Trials Unit, Nanjing Drum Tower Hospital, Affiliated Hospital of Medical School, Nanjing University, Nanjing, China; ^2^ Phase I Clinical Trials Unit, Nanjing Drum Tower Hospital, Nanjing Drum Tower Hospital Clinical College of Nanjing University of Chinese Medicine, Nanjing, China

**Keywords:** steroid hormone biosynthesis, liver regeneration, 2/3 partial hepatectomy, gut microbiota, metabolomics

## Abstract

**Introduction:** The liver is the only organ capable of full regeneration in mammals. However, the exact mechanism of gut microbiota and metabolites derived from them relating to liver regeneration has not been fully elucidated.

**Methods:** To demonstrate how the gut-liver axis contributes to liver regeneration, using an LC-QTOF/MS-based metabolomics technique, we examine the gut microbiota-derived metabolites in the gut content of C57BL/6J mice at various points after 2/3 partial hepatectomy (PHx). Compound identification, multivariate/univariate data analysis and pathway analysis were performed subsequently. The diversity of the bacterial communities in the gastrointestinal content was measured using 16S rRNA gene sequencing. Then, the integration analysis of gut microbiota and metabolome was performed.

**Results:** After 2/3 PHx, the residual liver proliferated quickly in the first 3 days and had about 90% of its initial weight by the seventh day. The results of PLS-DA showed that a significant metabolic shift occurred at 6 h and 36 h after 2/3 PHx that was reversed at the late phase of liver regeneration. The α and β-diversity of the gut microbiota significantly changed at the early stage of liver regeneration. Specifically, *Escherichia Shigella*, *Lactobacillus*, *Akkermansia*, and *Muribaculaceae* were the bacteria that changed the most considerably during liver regeneration. Further pathway analysis found the most influenced co-metabolized pathways between the host and gut bacteria including glycolysis, the TCA cycle, arginine metabolism, glutathione metabolism, tryptophan metabolism, and purine and pyrimidine metabolism. Specifically, steroid hormone biosynthesis is the most significant pathway of the host during liver regeneration.

**Discussion:** These findings revealed that during liver regeneration, there was a broad modification of gut microbiota and systemic metabolism and they were strongly correlated. Targeting specific gut bacterial strains, especially increasing the abundance of *Akkermansia* and decreasing the abundance of *Enterobacteriaceae*, may be a promising beneficial strategy to modulate systemic metabolism such as amino acid and nucleotide metabolism and promote liver regeneration.

## 1 Introduction

The liver is the largest organ of the mammal’s internal organs and is mainly responsible for maintaining internal balance, involving the elimination of waste, the storage and balancing of glucose and glycogen, the regulation of amino acid metabolism, detoxification, drug metabolism, and more. The liver is the only internal organ in mammals with the amazing capacity to regenerate to 100% of its initial weight, guaranteeing that the liver-to-weight ratio is consistently maintained at the level necessary for maintaining bodily homeostasis (Tremaroli and Bäckhed, 2012). Liver regeneration is a highly coordinated biological process. Many changes in gene and protein expression, the production of growth factor, and tissue remodeling occurred during this process. Multiple types of cells and extrahepatic factors cooperated during liver regeneration (Mao et al., 2014). Based on this characteristic of the liver, hepatic resection has been established as a safe primary treatment for a variety of diseases of the liver, such as hepatic trauma, intrahepatic gallstones, hepatic cysts, hepatic tumors, and liver transplantation (Rahbari et al., 2021); however, successful restoration of the patient’s hepatic function in the postoperative period is critical for their good prognosis and quality of life.

After hepatic resection, hepatocytes that have not undergone final differentiation show remarkable proliferative capacity (Liu et al., 2015), in addition to cytokines, growth factors and mitogens also participate in the process of hepatic regeneration. Energy production and the provision of precursors for the manufacture of chemicals essential for hepatic cell proliferation and tissue remodeling are dependent on active metabolism (Fausto et al., 2012; Michalopoulos, 2014; Monga, 2014). Liver regeneration is accompanied by changes in system metabolism. Metabolic change is a sign of various liver diseases, and liver regeneration after PHx is also characterized by a changed status of metabolism, in which the body undergoes significant changes in metabolites reflected in the serum, liver and urine (Kajiura et al., 2019). Metabolomics is a method of comprehensive and systematic analysis and quantitate of metabolites in biological matrix using high-throughput technologies. A number of studies have been conducted on rodent partial hepatectomy models with metabolomics techniques to explore the alteration of metabolism related to liver regeneration (Kajiura et al., 2019). These studies have pointed out that metabolic pathways including bile acid metabolism have an essential role in the whole complex process of liver regeneration (de Haan et al., 2018).

Gut microecology is essential in regulating metabolic activities (Tremaroli and Bäckhed, 2012; Cani, 2014), modulating immunity (Viaud et al., 2013) and protecting the host from pathogenic microorganisms, etc (Fukuda et al., 2011). Many diseases are at risk due to dysbiosis of the intestinal flora, and in this regard, the connection between the liver and the gut is becoming more well-recognized (Tripathi et al., 2018). The gut-liver axis, which is the integrated functional and physiological relationship between the liver and the gut, is the focus of current research. The intestinal flora is validated can influence the pathophysiology of the liver through the gut-liver axis. Numerous liver illnesses, such as alcoholic liver disease and nonalcoholic fatty liver disease, primary sclerosing cholangitis, and hepatocellular carcinoma, etc. are linked to changes in the composition of the gut microbiota. In addition, gut flora has been shown to influence the regenerative capacity of the liver. Studies have shown that the gut microbiota acts through the gut-liver axis to link a wide range of metabolites to liver regeneration and that these metabolites are important signaling and energy substrates for hepatocytes (Yin et al., 2023). A shift in the gut microbiota ratio results in modifications to the metabolites, which in turn affect hepatocyte proliferation and metabolism, thus reducing liver injury and enhancing liver regeneration (Liu et al., 2016). In general, the etiology of liver disease and the process of liver regeneration are significantly influenced by gut bacteria.

With the development of metabolomics and advanced bioinformatics analysis, the relationship between the functional activity of the gut microbiota and changes in endogenous metabolites [bile acids (Huang et al., 2006), short-chain fatty acids (Yin et al., 2023), tryptophan (Xu et al., 2022), etc.] and liver regeneration has been reported; yet, the connection between the gut microbiota and the metabolites derived directly from them and liver regeneration has not been fully elucidated. In this study, gut microbiota and metabolites in the gut content were analyzed in an integrated manner, and this comprehensive and integrated approach may provide a clearer understanding of the function of gut microbiota and metabolites derived from them and the underlying mechanisms of liver regeneration.

## 2 Methods

### 2.1 Animals and treatment

Thirty male C57BL/6J mice, 6 weeks old, were kept in a 12 h light/12 h dark environment (lights on at 6:00 a.m. and lights off at 6:00 p.m.). The mice were acquired from Changzhou Cavens Laboratory Animal Co. (Changzhou, China). The Animal Ethics Committee of Nanjing University Medical School Affiliated Drum Tower Hospital accepted all protocols pertaining to animal care and experimental procedures. Five groups (n = 6) of mice were used: the Sham group (PHx-0h), the PHx-6h group, the PHx-36h group, the PHx-72h group, and the PHx-168h group. The PHx surgery was performed as previously reported (Kong et al., 2018). Briefly, the mice were anesthetized with isoflurane, the mouse abdomen was skinned and disinfected, then fully exposes the liver and ligate the roots of the left and middle lobes of the liver with 5-0 silk thread respectively and then both lobes are removed. The control group (sham operation group) only underwent laparotomy and suturing. The mice were sacrificed at different time points after PHx. Serum and tissues were collected and used for further analysis. The detailed information can be found in Supplementary Material.

### 2.2 Untargeted metabolomics of the gut content by LC-QTOF/MS

The same extraction and analysis procedures were applied as previously reported with a few adjustments (Sun et al., 2023a; Sun et al., 2023b). To summarize, 20 mg of gut material was added to 100 μL of normal saline, and 50 μL of the gut content combination was then mixed with 200 μL of methanol that contained 1 μg/mL of 4-chlorophenylalanine as an internal standard (IS). Following a 3-min vortex and 10 min of centrifugation at 12,000 rpm, 200 μL of the supernatant was moved to a fresh Eppendorf tube and dried using an SPD 2010-230 SpeedVac Concentrator (Thermo Savant, Holbrook, United States). The residue was re-dissolved with 100 μL 50% methanol-water and centrifuged at 18,000 rpm for 10 min. Finally, 80 μL supernatant was transferred to an LC vial and 5 μL was injected and analysed. The chromatographic and mass spectrometric parameters were the same as reported before (Sun et al., 2023b). Briefly, the LC-Q/TOF-MS (AB SCIEX TripleTOF^®^ 5600 LC-Q/TOF-MS, Foster City, CA) was used for the untargeted metabolomics analysis both in positive and negative mode. TOF MS ranged from m/z 50–1,200. Then compound identification, multivariate and univariate data analysis were performed. Pathway analysis was carried out using the online metabolomics data analysis tool MetaboAnalyst 6.0 (www.metaboanalyst.ca) with the KEGG library (*Mus musculus*). Enrichment analysis was also performed using chemical structures. The detailed information can be found in Supplementary Material.

### 2.3 16S rDNA gene sequencing

The 16S rDNA gene amplicon sequencing of gut microbiota was performed by Suzhou PANOMIX Biomedical Tech Co. Ltd. (Suzhou, China) using an Illumina MiSeq platform (Illumina, San Diego, CA, United States) (Sun et al., 2023b). The detailed information can be found in Supplementary Material.

### 2.4 Integration analysis of microbiome and metabolome

The integration analysis of the microbiome and metabolome was conducted using metorigin (https://metorigin.met-bioinformatics.cn) (Yu et al., 2022). *Mus musculus* (house mouse) was selected as the host.

### 2.5 Statistical analysis

Statistical analysis was performed using the R project (version 4.2.1) and GraphPad Prism 7.2 software (La Jolla, CA, United States). The data was displayed as mean ± standard deviation. A one-way ANOVA was used to examine group differences, and the Bonferroni *post hoc* test was used afterward. A significance level of *P* < 0.05 was applied to all statistical tests.

## 3 Results

### 3.1 The liver regenerated to normal after 2/3 PHx in mice

**FIGURE 1:**
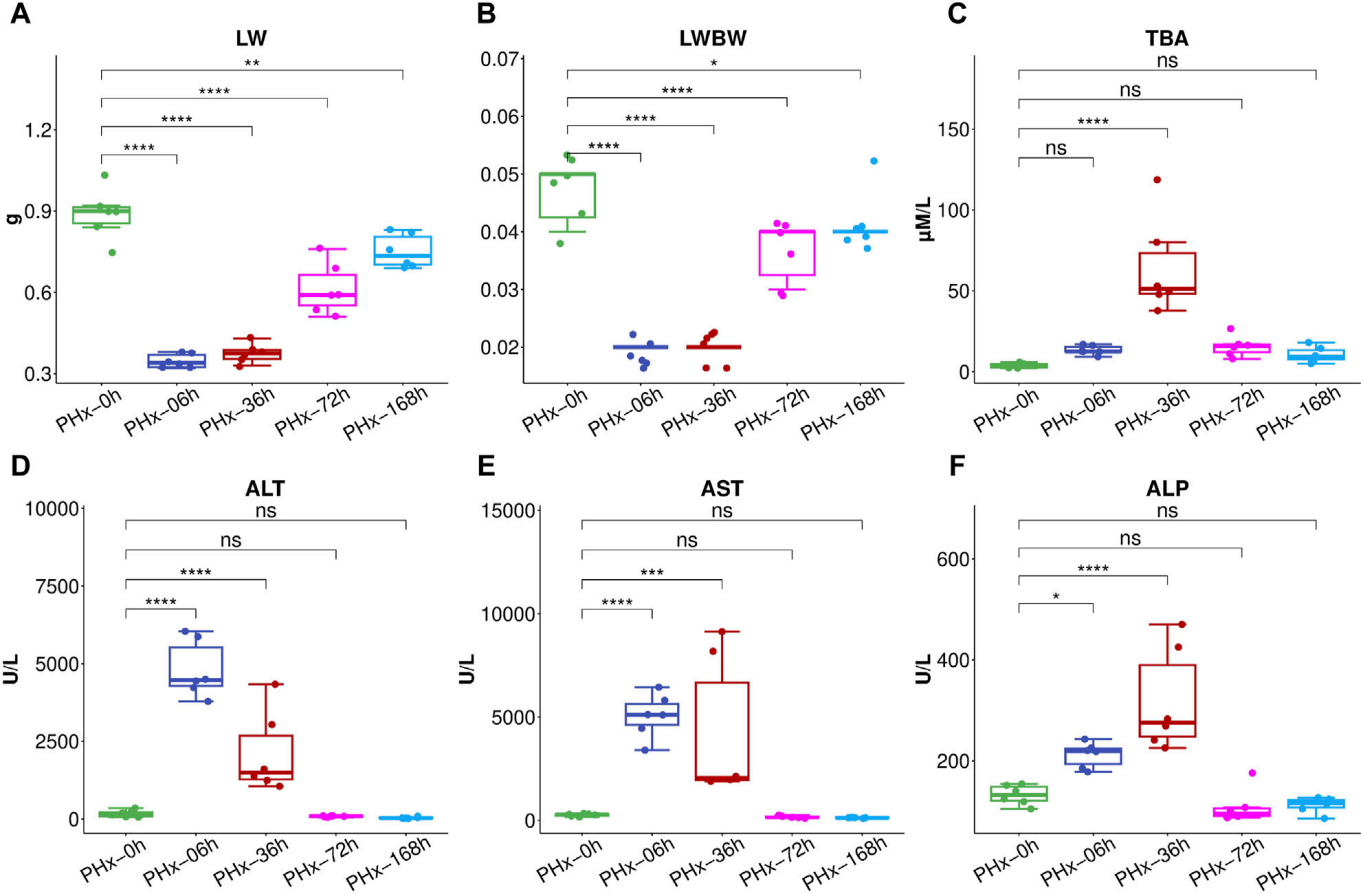
After 2/3 PHx, liver weight **(A)** and liver index **(B)** at various phases. The following biomarkers were assessed for the Sham group and 36, 72, and 168 h after PHx: total bile acids (TBA) **(C)**, Alanine aminotransferase (ALT) **(D)**, aspartate aminotransferase (AST) **(E)**, alkaline phosphatase (ALP) **(F)**. The data (n = 6) are displayed as mean ± SD. *** *P* < 0.001, ** *P* < 0.01 and * *P* < 0.05.

Serum and liver samples were collected at five time points (0 h, 6 h, 36 h, 72 h, and 168 h) after surgery to inquire about the changes in the gut microbiota and the metabolites during liver regeneration progresses following 2/3 PHx. According to the liver index, the remaining liver grew more quickly in the first 3 days and then reached over 90% of its initial weight again on the seventh day (Figures 1A, B). Following 2/3 PHx, the serum’s total bile acids (TBA) considerably rose in the early stages (Figure 1C). ALT (Figure 1D), AST (Figure 1E), and ALP (Figure 1F) were significantly increased during the early stages of liver regeneration and recovered to normal after 72 h.

### 3.2 A significant metabolic change in the gut content occurred after 2/3 PHx

**FIGURE 2:**
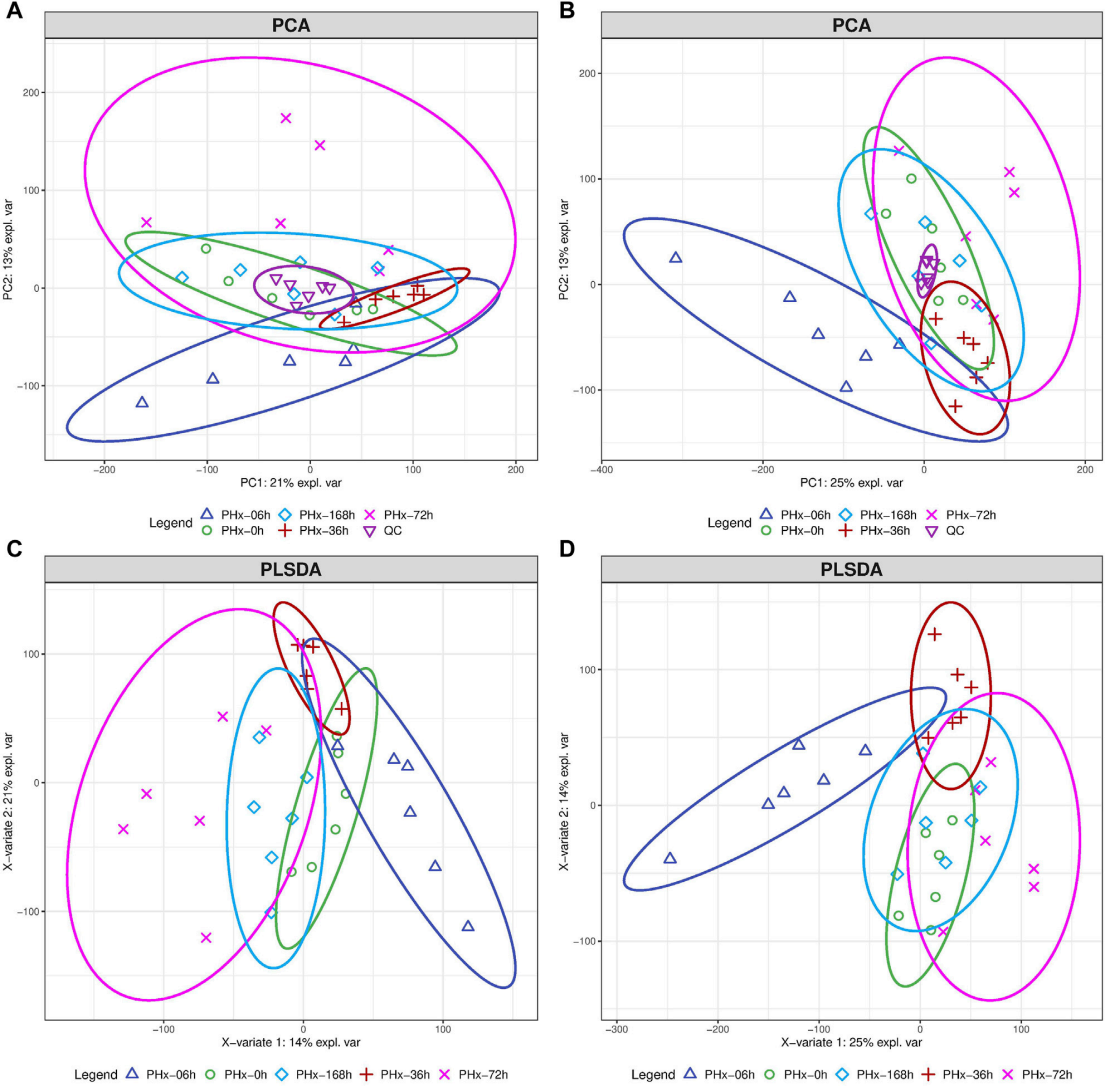
The total alteration of metabolites at different phases following a 2/3 PHx. The score plots for PCA model of mouse gut content at positive mode **(A)** and negative mode **(B)**. Score plots for the PLS-DA model’s positive mode **(C)** and negative mode **(D)**.

The typical total ion current (TIC) chromatograms of mouse gut content in positive and negative modes are shown in Supplementary Figure S1. 23,225 and 26,524 chromatographic features were deconvoluted and extracted respectively. The compounds identified include organic acids, amino acids, carbohydrates, purines, pyrimidines and fatty acids. An overview of the metabolomics data was obtained using unsupervised principal component analysis (PCA). In the PCA analysis, no outlier was discovered based on the scatter plots (Figures 2A, B). The partial least squares discriminant analysis (PLSDA) showed that the 6 h group, the 36 h group, and the Sham group were clearly separated from one another, while the 72 h group and the 168 h group were closer to the Sham group (Figures 2C, D). This suggests that during the liver regeneration process, PHx induced a significant metabolic change in the gut content at an early stage and returned to normal at the late stage.

### 3.3 Metabolites in the gut content changed significantly during liver regeneration after 2/3 PHx

To select the differential metabolites in the gut content during liver regeneration after 2/3 PHx, the average of each of the two groups was divided to determine the fold change (FC), and the Benjamini–Hochberg method was used to adjust the Student’s t-test to determine the *P*-value. Metabolites in the gut contents with an FC of greater than 2 or less than 0.5 and a *P*-value of less than 0.05 are considered to be differential metabolites and displayed in the volcano plot (Supplementary Figures S2A–D, positive mode; -Supplementary Figures S3A–D, negative mode). The heat maps of differential metabolites in the gut contents of the positive and negative modes are shown in Supplementary Figures S4, S5, respectively. Specifically, metabolites in TCA cycle (succinic acid), nucleotides (cAMP, AMP, Uridine), amino acids (L-cystine), the steroid hormones (cortisone, corticosterone, deoxycorticosterone and 17-hydroxyprogesterone) all peaked at the early phase (6 h or 36 h) during liver regeneration after 2/3 PHx, and returned to normal at the late phase (Supplementary Figure S6).

### 3.4 Energy metabolism, amino acid metabolism and nucleotide metabolism are the significantly changed metabolic pathways during liver regeneration after 2/3 PHx

The significantly changed metabolites in the gut content were selected and further analyzed by MetaboAnalyst (www.metaboanalyst.ca). In order to analyze the metabolites of gut contents for overrepresentation and pathway analysis, the differential metabolites were mapped to KEGG metabolic pathways.

**FIGURE 3:**
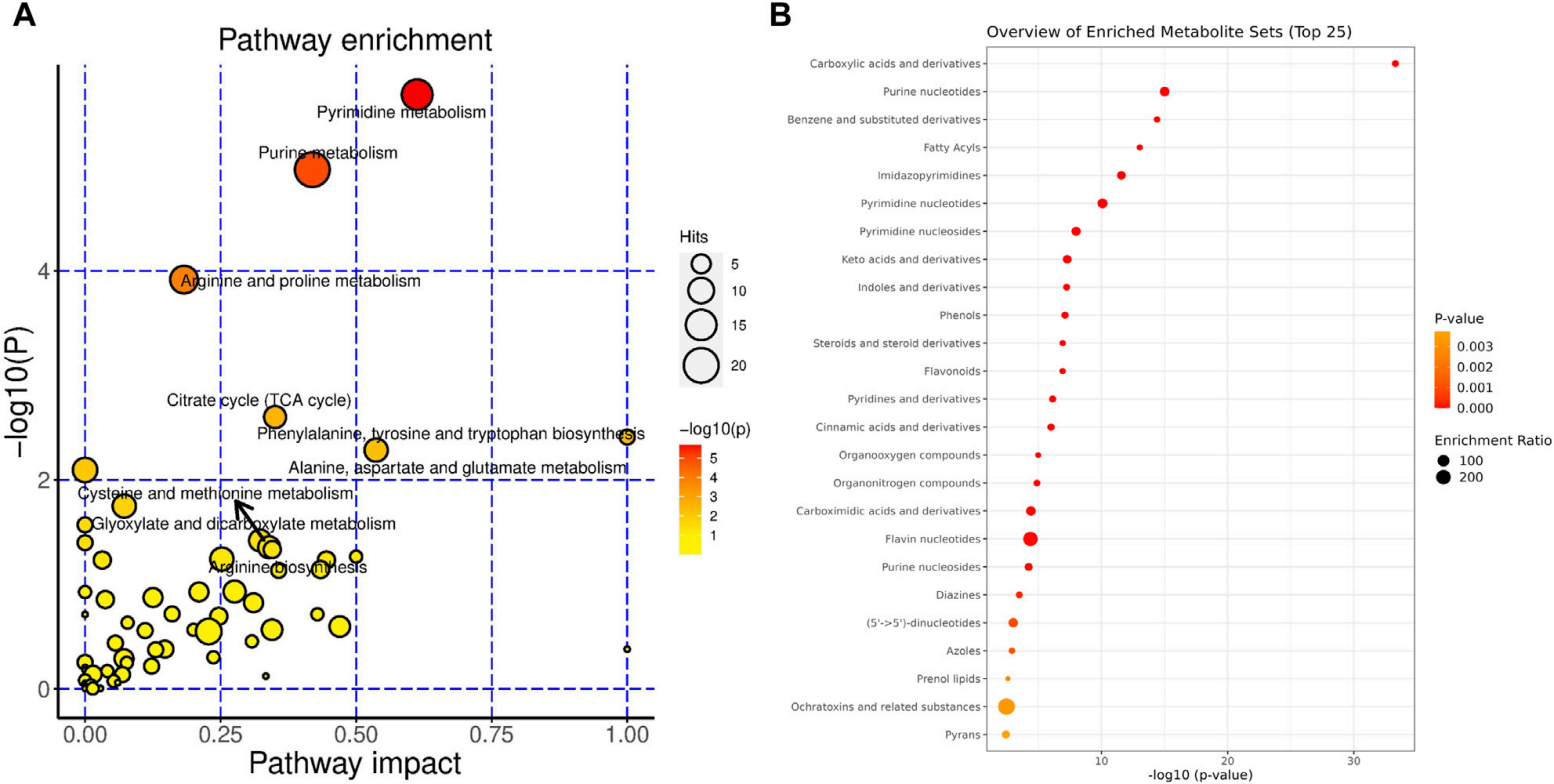
Metabolites in the gut content significantly changed during liver regeneration after 2/3 partial hepatectomy. Metabolic pathways that are modulated considerably during liver regeneration: **(A)**. The classification of metabolites that are significantly modulated during liver regeneration **(B)**.

The pathway analysis showed that glyoxylate and dicarboxylate metabolism, the citrate cycle (TCA cycle), alanine, aspartate and glutamate metabolism, cysteine and methionine metabolism, purine metabolism and pyrimidine metabolism were the most changed pathways (Figure 3A). Accordingly, the enrichment analysis showed that the most enriched metabolites set including carboxylic acids and derivatives, purine nucleotides/nucleotides, pyrimidine/nucleotides, benzene and substituted derivatives, steroids and steroid derivatives, indoles and derivatives and fatty acyls etc (Figure 3B).

### 3.5 Alterations of gut microbial diversity and abundance during liver regeneration after 2/3 PHx

**FIGURE 4:**
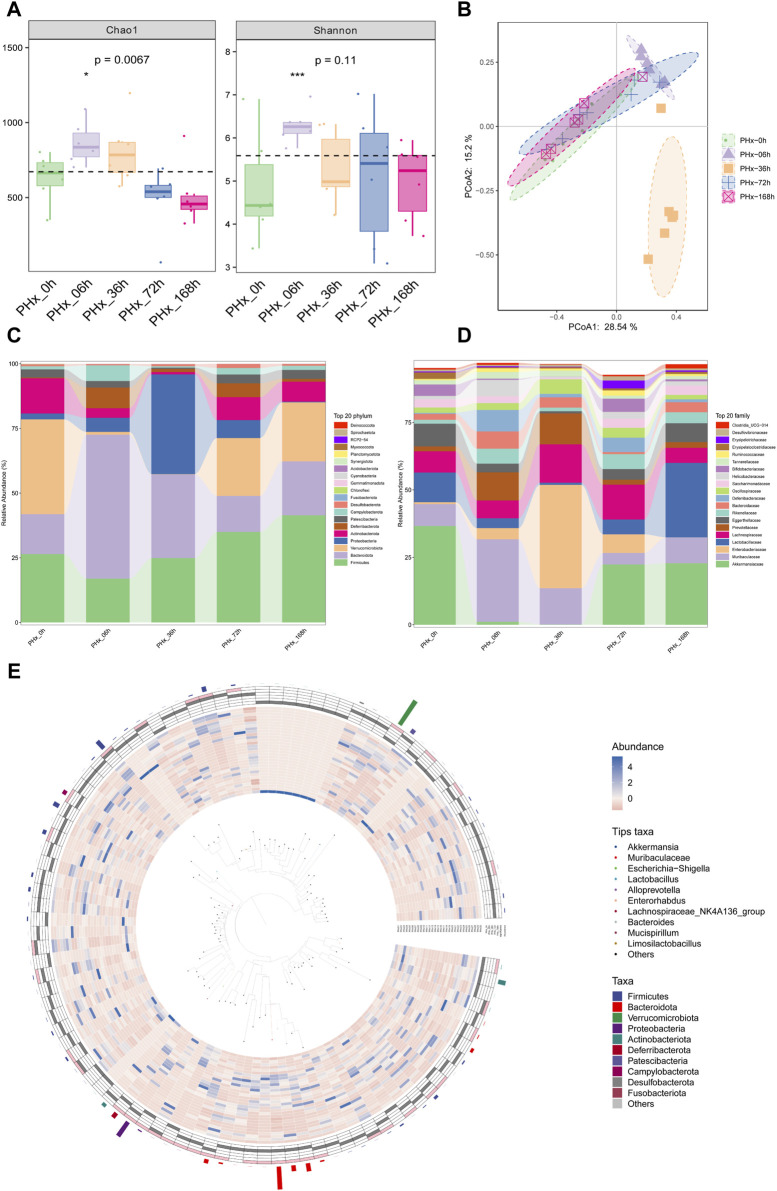
Alterations in composition of gut microbiota during liver regeneration after 2/3 partial hepatectomy. **(A)** α diversity of gut microbiota (Chao1 and Shannon index). **(B)** PCoA of β diversity. **(C)** Alteration of gut microbiota composition at phylum levels. **(D)** Alteration of gut microbiota composition at family levels. **(E)** Graphical Phylogenetic Analysis (GraPhlAn) Phylogenetic tree.

16S rRNA sequencing was used to investigate the bacterial communities. The α diversity of gut microbiota is shown in Figure 4A. The Chao1 index and the Shannon index showed that the α diversity of the gut bacteria increased significantly. The PCoA results representing the β diversity (Figure 4B) showed that the composition of microbial communities at various times after 2/3 PHx was significantly separated, indicating a dramatic alteration of the microbial composition. The considerable distance between the PHx-6 h group and the PHx-36 h group with the sham group suggested that the abundance of the microbial community could be obviously modulated during liver regeneration, and the changed abundance of the microbial will return at the late phase of liver regeneration.

The top 20 most prevalent bacteria at the phylum level and family level in the gut contents are presented in Figures 4C, D, respectively. Specifically, compared with the Sham group, At the early phase, PHx markedly decreased the relative abundance of *Firmicutes* and *Verrucomicrobia* and increased the relative abundances of *Bacteroidetes* and *Proteobacteria* at the genus level. At the family level, PHx markedly decreased the relative abundance of *Akkermansiaceae* and *Lactobacillales* and increased the relative abundances of *Muribaculaceae* and *Enterobacteriaceae* at the early phase. These changes all returned to normal at the late phase of PHx. Figure 4E shows the biological evolution tree of each group.

**FIGURE 5:**
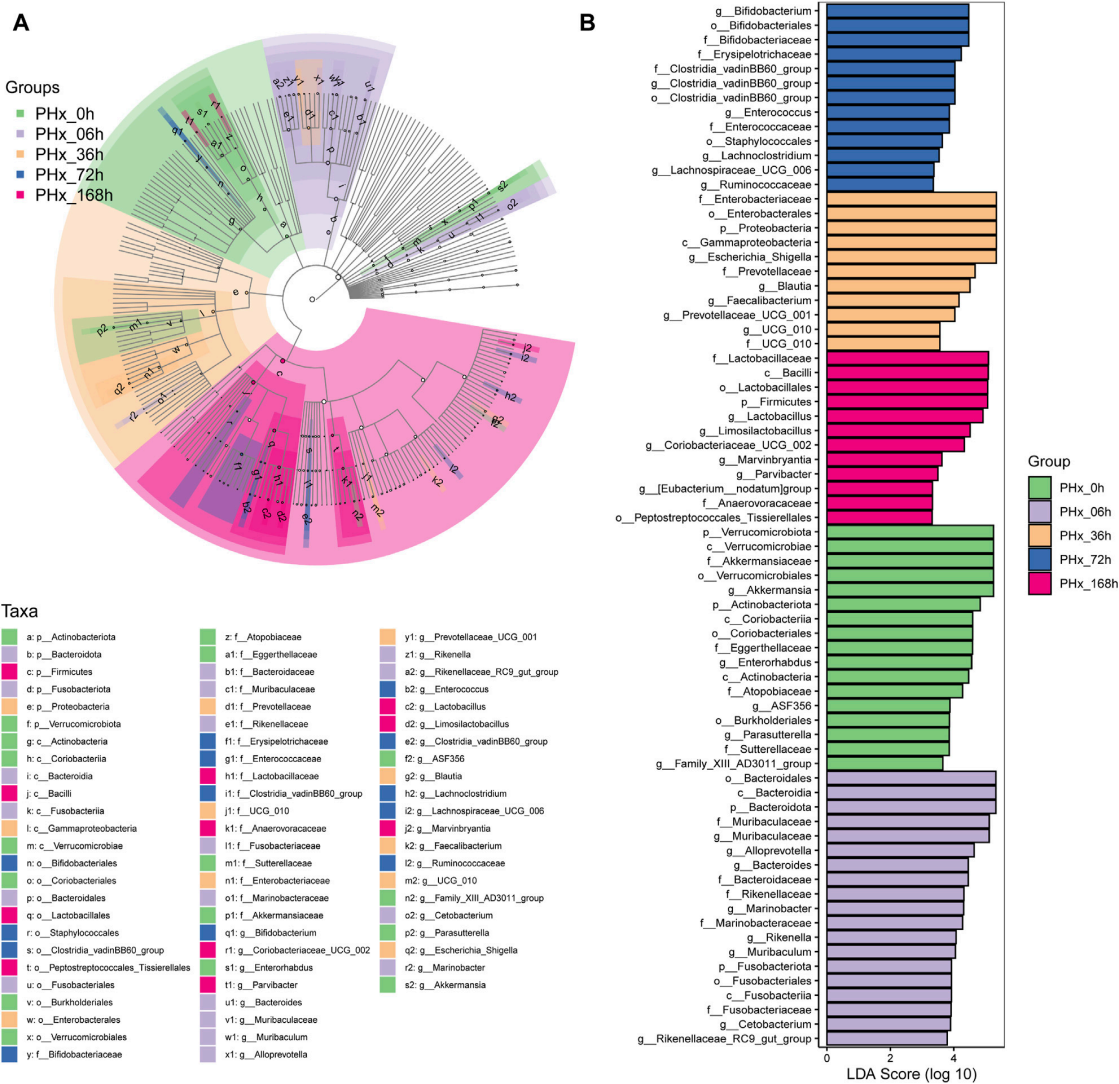
Identifying the distinctive taxa that differ the most during liver regeneration following a 2/3 PHx. **(A)** Characteristic taxa are analyzed using linear discriminant analysis (LDA) effect size (LEfSe) across five experimental groups. **(B)** Use LDA to characterize taxa with a threshold score greater than 3.0. The influence of distinctive taxa within specific groups is shown by the bar length of the LDA.

LefSe was also used to evaluate the species and relative abundance of gut microbiota in each group. Following PHx, the evolutionary branch diagram displayed the most distinct bacteria at each time point (Figure 5A). The results (Figure 5B) showed that at the phylum level, *Verrucomicrobiota*, *Bacteroidota*, *Proteobacteria*, and *Firmicutes* are the most differentially enriched bacteria in the 0 h, 6 h, 36 h, and 168 h groups, respectively. At the class level, *Verrucomicrobiae*, *Bacteroidia*, *Gammaproteobacteria* and *Bacilli* are the most differentially enriched bacteria in the 0 h, 6 h, 36 h, and 168 h groups, respectively. At the order level, *Verrucomicrobiales*, *Bacteroidales*, *Enterobacterales*, *Bifidobacteriales* and *Lactobacillales* are the most differentially enriched bacteria in the 0 h, 6 h, 36 h, 72 h, and 168 h groups, respectively. At the family level, *Akkermansiaceae*, *Muribaculaceae*, *Enterobacteriaceae*, *Bifidobacteriaceae* and *Lactobacillaceae* are the most differentially enriched bacteria in the 0 h, 6 h, 36 h, 72 h, and 168 h groups, respectively. At the genus level, *Akkermansia*, *Muribaculaceae*, *Escherichia Shigella*, *Bifidobacterium* and *Lactobacillus* were the most differentially enriched bacteria in the 0 h, 6 h, 36 h, 72 h, and 168 h groups, respectively.

### 3.6 Gut microbiota and metabolites derived from them were closely related

**FIGURE 6:**
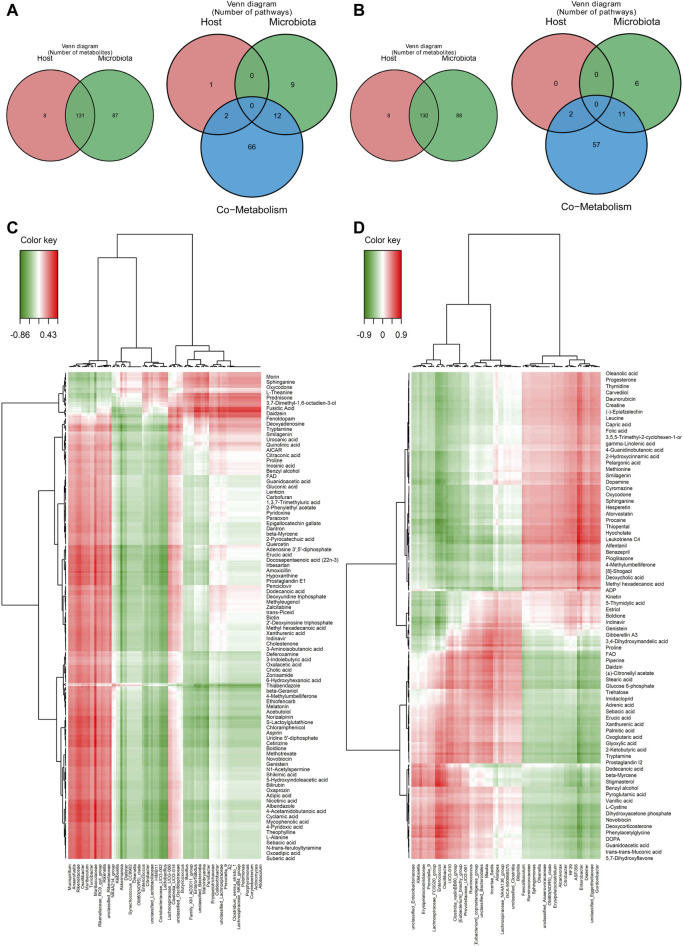
Correlation study between the gut microbiota and the considerably altered metabolites in the gut content. **(A)** Metabolites and pathways in the microbiota and host significantly altered 6 hours after 2/3 PHx. **(B)** Metabolites and pathways in the microbiota and host significantly altered 36 h after 2/3 PHx. **(C)** A correlation heatmap showing the major metabolite changes in the gut microbiota and gut content 6 h and after 2/3 PHx. **(D)** A correlation heatmap showing the metabolites that were significantly altered in the gut microbiota and gut content 36 h after 2/3 PHx.

The correlation between the gut microbiota and metabolites derived by them was analyzed, and the metabolites and pathways in the host and microbiota respectively and co-metabolized by the host and microbiota were shown in the venn plot (Figures 6A, B). A total of 130 metabolites and two pathways were co-metabolized by the host and microbiota. Spearman’s correlation analysis between the metabolites in the gut contents and the differential bacteria at the genus level was performed in order to better evaluate the possible association between the gut microbiota and metabolites derived by them (Supplementary Figure S7). The correlations of gut bacteria and metabolites were shown in Figures 6C, D for 6 h and 36 h after PHx, respectively. Specifically, at 6 h, morin, sphinganine, oxycodone, L-theanine and prednisone, etc. were negatively correlated with *Mucispirillum*, *Anaerofustis*, *Rikenellaceae*, *Oscillibacter* and *Muribaculum*, whereas positively correlated with *Pelomonas*, *Porphyromonas*, *Corynebacterium*, *Actinomyces* and *Allobaculum*. L-alanine, sebacic acid, N-trans-feruloyltyramine, oxoadipic acid and suberic acid, etc., have the opposite trend with these bacteria (Figure 6C). At 36 h, oleanolic acid, progesterone, thymidine, carvedilol and creatine were negatively correlated with *Klebsiella*, *Erysipelatoclostridiaceae*, *Prevotella 9*, *Enterococcus* and *Oscillibacter*, and positively correlated with *RF39*, *ASF356*, *Enterobacter*, *Dialister* and *Gordonibacter*. Deoxycorticosterone, phenylacetylglycine, DOPA, guanidoacetic acid, trans-muconic acid and 5,7-Dihydroxyflavone etc. have the opposite trend with these bacteria (Figure 6D).

### 3.7 Network analysis of gut microbiota and metabolites derived by them revealed that steroid hormone biosynthesis is the most important altered metabolic pathway in the host

**FIGURE 7:**
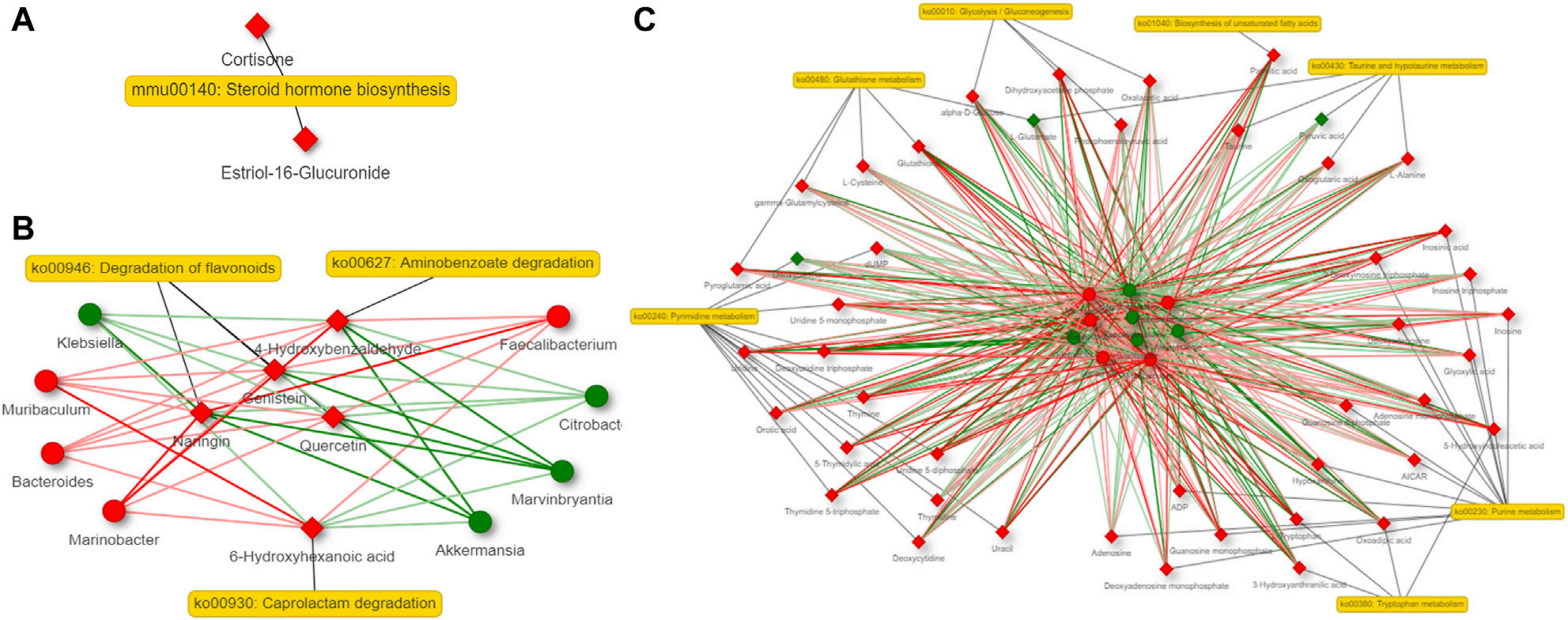
Network analysis of host metabolites **(A)**, bacteria metabolites **(B)** and co-metabolism **(C)** at 6 h after 2/3 PHx. Microbes are shown by round nodes, and metabolites are shown by cubic nodes.

**FIGURE 8:**
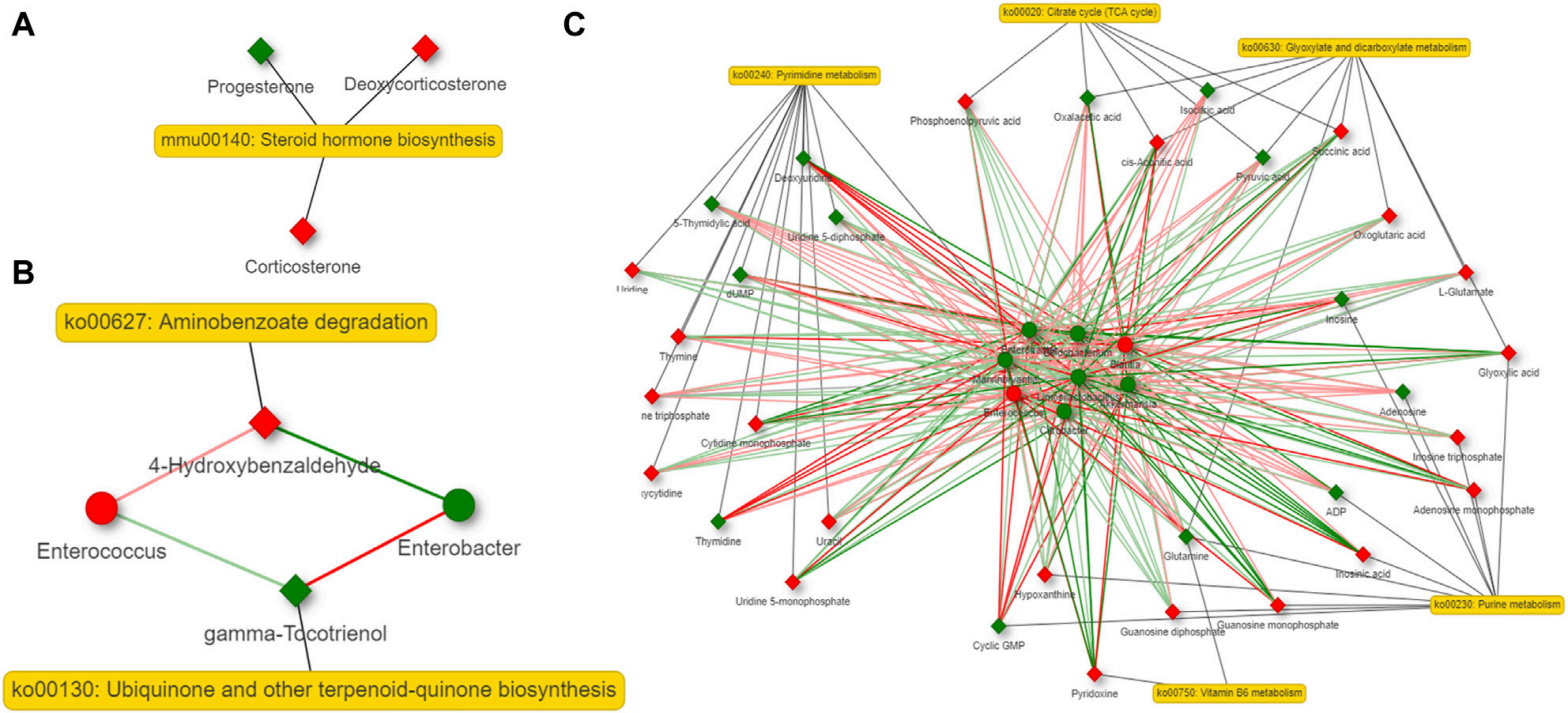
Network analysis of host metabolites **(A)**, bacteria metabolites **(B)** and co-metabolism **(C)** at 36 h after 2/3 PHx. Microbes are shown by round nodes, and metabolites are shown by cubic nodes.

A co-occurrence network analysis among the differential microbiota and metabolites derived by them was carried out by contrasting the 6 and 36-h groups with the Sham group. Steroid hormone biosynthesis is the most important altered metabolic pathway in the host. Aminobenzoate degradation, degradation of flavonoids and caprolactam degradation are the most important changed metabolic pathways in the bacteria at 6 h after 2/3 PHx. Aminobenzoate degradation and ubiquinone and other terpenoid-quinone biosynthesis are the most important changed metabolic pathways in the bacteria at 36 h after 2/3 PHx. The TCA cycle, glycolysis, tryptophan metabolism, glutathione metabolism, taurine and hypotaurine metabolism, biosynthesis of unsaturated fatty acids, purine and pyrimidine metabolism were the important metabolic pathways co-metabolized by the host and bacteria at 6 h after 2/3 PHx (Figure 7). Glyoxylate and dicarboxylate metabolism, the TCA cycle, vitamin B6 metabolism, purine and pyrimidine metabolism were the important metabolic pathways co-metabolized by the host and bacteria at 36 h after 2/3 PHx (Figure 8). These results suggested that gut microbiota and metabolites derived by them dynamically changed during liver regeneration.

## 4 Discussion

The complicated biological process of liver regeneration involves successive alterations in cytokines, metabolites, and gene expression, among others. After 2/3 PHx, gut-liver axis interaction is crucial to liver regeneration. Gut microbiota plays a key role in the gut-liver axis and influences many areas of human health and diseases. It is also found to be essential in liver regeneration. Liver regeneration is delayed in Germ-free mice and after the use of antibiotics in normal mice (Cornell et al., 1990; Wu et al., 2015). Reconstitution of normal gut microbiota in GF mice could ameliorate liver regeneration (Amin et al., 2020). Several studies also demonstrate the beneficial effects of probiotics and specific gut microbiota on the promotion of liver regeneration (Xie et al., 2021; He et al., 2023). By stimulating the phospholipid biosynthesis of the hepatic membrane, gut bacteria may contribute to liver regeneration (Yin et al., 2023). These findings demonstrate how crucial gut microbiota is to liver regeneration. However, the underlying mechanisms of gut-liver axis interaction involving gut microbiota in liver regeneration are not fully understood, and the modulation of gut microbiota and the metabolites derived from them after PHx and additional investigation are still required to determine the underlying mechanisms. In this study, we discovered that there was a notable shift in the gut microbiota’s composition at different stages during liver regeneration, especially at 6 h and 36 h after PHx. The metabolites in the gut content also showed an obvious alteration at various time points after 2/3 PHx. The correlation between gut microbiota and metabolites derived by them showed that several metabolic pathways are changed during liver regeneration, including pathways in host-specific metabolism, microbiota-specific metabolism and host and microbiota co-metabolized.

Our study showed the alteration of gut microbial composition after PHx by 16S rRNA sequencing. The process of liver regeneration causes a dynamic shift in the composition of the gut flora. The α and β diversity of the gut bacteria both changed. This is in line with other research (Liu et al., 2016). Specifically, at the genus level, PHx markedly decreased *Firmicutes* and *Verrucomicrobia* and increased *Bacteroidetes* and *Proteobacteria*. The ratio between *Firmicutes*/*Bacteroidetes* is reported to be associated with obesity, diabetes, NAFLD/NASH, inflammatory bowel disease and even cancer. The elevated F/B ratio that happened within the first 6 h after PHx might be more efficient in producing energy for the regenerative liver. *Muribaculaceae*, *Prevotellaceae*, *Rikenellaceae* and *Bacteroidaceae* were the most predominant family in the *Bacteroidetes* phylum, whereas *Lactobacillaceae*, *Lachnospiraceae*, *Oscillospiraceae* and *Ruminococcaceae* were the most prevalent members of the phylum *Firmicutes*. These bacteria can provide energy, and have the ability to produce several metabolites and cytokines that shape the gut environment, maintain intestinal barrier function and be beneficial for maintaining gut health. Thus, we hypothesized that these bacteria were involved in modulating the metabolites in the body and promoting liver regeneration (Grigor’eva, 2020; Kusnadi et al., 2023; Stojanov et al., 2020; An et al., 2023).

An important factor in liver regeneration is metabolism. Glucose, triglycerides, cholesterol, and amino acids were significantly changed during liver regeneration (Bollard et al., 2010; Huang and Rudnick, 2014; Caldez et al., 2018). Consistently, metabolic genes are altered during liver regeneration. Through the portal vein, the body absorbs metabolites from the gut microbiota, which serve as energy substrates and vital signaling molecules. Lipopolysaccharide (LPS), bile acids, short-chain fatty acids (SCFAs) and tryptophan metabolites, etc. were the main metabolites produced by gut microbiota. Several studies have revealed the multifaceted roles of these metabolites in liver regeneration. The appropriate amount of LPS, the major component of the outer wall of the Gram-negative bacteria, is beneficial for liver regeneration, mainly by promoting hepatocyte DNA replication (Zheng and Wang, 2021). SCFAs (acetic acid, propionic acid and butyric acid) are produced by gut microbiota during the degradation of dietary fiber. SCFAs are considered energy sources for host colonocytes, they can also improve the metabolism homeostasis, shape the gut environment and maintain intestinal barrier function, thus promoting liver regeneration (Ríos-Covián et al., 2016; Murugesan et al., 2018). In our study, these metabolites were not detected. This is because LPS has a molecular weight of 10–20 kDa and can not be detected by LC-TOF/MS. Similarly, SCFAs also can not be detected because they are small polar volatile compounds and GC/MS is more suitable for detecting them. In addition, the functions of these metabolites have been well elucidated. Thus we mainly focus on the metabolites including amino acids, organic acids, purine and pyrimidine nucleotides and lipids, etc.

Our results show obvious alteration of metabolites in these pathways, including glycolysis, the TCA cycle, amino acid metabolism, purine and pyrimidine metabolism, etc. Consequently, we deduced that these pathways contribute significantly to the metabolic process involved in liver regeneration. These metabolic pathways were basic life activities and performed both by the host and bacteria. However, it’s difficult to determine whether these metabolites were produced by host or bacterial. Enzymes produced by the gut microbiota known as microbial-host isozymes operate similarly to host enzymes and may be involved in gut metabolism and preserving host metabolic homeostasis (Wang et al., 2023). We speculated that the microbial-host-isozymes also changed during liver regeneration and contributed to the alteration of metabolites.

Glycolysis and the TCA cycle are metabolic pathways that generate energy. Additionally, they provide the intermediary molecules needed for additional pathways. Pyruvate, lactate and succinate, etc. can be converted into acetate, propionate and butyrate by the gut microbiota. They appear to be the natural ligands of G-protein-coupled receptor (GPR), activate the downstream signaling pathways, improve the metabolism homeostasis and promote liver regeneration (Portincasa et al., 2022). According to these findings, these metabolic intermediates modulate energy metabolism and liver regeneration in a significant way. This effect may be due to the control of intestinal cells as well as the gut microbiota.

Amino acids are the building blocks of protein. Protein synthesis is necessary for cell proliferation, which allows cells to grow and divide rapidly during liver regeneration. The liver has a pivotal role in amino acid metabolism. Besides, amino acids also function as signaling molecules and participate in many essential critical cellular functions. We found that arginine metabolism, taurine and hypotaurine metabolism, and glutathione metabolism were identified to be attributable to liver regeneration. Glutathione is a critical intracellular antioxidant, its concentration was doubled after PHx, which can maintain immune system homeostasis and activate hepatic NF-κB to promote liver regeneration (Irino et al., 2016). Taurine is a sulfur-containing amino acid, it can conjugate with bile acids to increase their aqueous solubility and decrease their cytotoxicity (Murakami et al., 2016). Taurine is also involved in many physiological functions including antioxidation, calcium modulation, and membrane stabilization. Taurine and hypotaurine metabolism is a pathway that is enriched during liver regeneration, consistent with the change of bile acids. The taurine-conjugated bile acid, taurocholic acid, can reduce the expression of connective tissue growth factor in hepatocytes through ERK-YAP signaling (Yu et al., 2018), and YAP is reported to be a core transcription coregulator in controlling organ growth including liver size (Jiang et al., 2019). The oxidative metabolite of L-arginine, nitric oxide (NO), plays a crucial role as a modulator of hepatocyte proliferation during liver regeneration. L-glutamine, a precursor for molecules including glutathione, nucleotides, and nucleic acids, can also function as a signaling molecule. L-arginine, L-glutamine, and BCAA supplements assist in speeding the recovery process of the liver following a hepatectomy (Kurokawa et al., 2012; Ceyhan et al., 2019).

Nuclear bases of nucleic acids, purine and pyrimidine nucleotides, are also important energy carriers. They also serve as building blocks for the synthesis of SAM, NAD, and other nucleotide cofactors. After PHx, the liver immediately starts to restore itself, which is a process that needs massive nucleotides, amino acids and energy. Nucleotide metabolism is a key metabolic process after PHx in the synthesis of DNA and RNA. Pyrimidine nucleoside uridine is found to be a potent regeneration-promoting factor (Liu et al., 2022). Nucleotides are also co-metabolized by the host and gut microbiota. The catabolism of purine nucleotides in the regenerating liver is accelerated and the pyrimidine pool is enlarged during liver regeneration (Usami et al., 1996). In our study, the purine and pyrimidine nucleotides were also significantly changed, these results suggested that PHx modifies the metabolism of pyrimidines and purines. A preoperative supply of nucleoside is effective for increasing hepatocyte DNA synthesis and hepatic regeneration after PHx (Usami et al., 1996).

According to earlier research, the body’s endogenous metabolites and gut bacteria are crucial for liver regeneration. Different from these studies, our study mainly focuses on the metabolites in the gut content, which we think can demonstrate the direct relationship that exists between the host’s gut flora and itself, and further reveal the function of gut microbiota-derived metabolites in liver regeneration. We further analyzed the results by correlation analysis to reveal the metabolites produced by the host and the gut microbiota, or co-metabolized. Our results revealed that steroid hormone biosynthesis is the most significantly changed pathway during liver regeneration, which is a host-specific metabolic pathway. Besides, bile acid metabolism is also enriched as a changed pathway. Thus we speculate that cholesterol metabolism, including primary bile acid biosynthesis and steroid hormone biosynthesis are important metabolic pathways during liver regeneration. Steroid hormones and bile acids (BAs) are both steroid molecules produced by the degradation of cholesterol. Cholesterol is an essential substance for liver recovery after PHx. It was metabolized to primary bile acids in hepatocytes and converted into secondary bile acids by the gut microbiota. In many liver illnesses, the metabolism of primary bile acid is a crucial pathway. BAs break down fats and help absorb them into the body. What’s more, they also have variate functions as signaling molecules. BAs are reported to be involved in the proliferation phase of liver regeneration. The right dosage of BAs may promote the growth of hepatocytes and cause the regeneration of the liver. However, the liver is harmed by too many BAs. Steroid hormones are found to be differential metabolites and the pathway was enriched in our study, which has not been reported by the previous studies. The role of steroid hormones in liver regeneration is complex. The steroid hormones progesterone, dehydroepiandrosterone (DHEA) (Kitamura et al., 1999), methandrastenolone, norethanedrolon, testosterone, and deoxycorticosterone were reported to accelerate this process (Gershbein, 1967). A low dose of dexamethasone ameliorates the transient impairment of liver regeneration, although it can not accelerate liver regeneration (Shibata et al., 2012). Controversially, cortisone, hydrocortisone and prednisolone depressed liver regeneration (Gershbein, 1973). Aldosterone, fludrocortisone, methylprednisolone have no apparent effects on hepatic regeneration (Glanemann et al., 2004). From a meta-analysis, pre-operative steroids may have a clinically significant benefit in liver regeneration (Richardson et al., 2014). The reason for the alteration in steroids and their function and mechanism in liver regeneration need to be further investigated.

The aminobenzoate degradation pathway was the most significant gut microbiota-specific metabolic pathway in the gut content of mice during liver regeneration. It was the third-largest xenobiotic biodegradation pathway by gut bacteria. This pathway can be cross-regulated between aerobic and anaerobic pathways of catabolized aromatic compounds, thus increasing the cell fitness in organisms, especially the gut that survive in environments subject to changing oxygen concentrations (Valderrama et al., 2012; Sridharan et al., 2014). 4-hydroxybenzaldehyde is an intermediate derived from tryptophan and other indole ring-containing compounds (Rudney and Parson, 1963). It was reported that 4-hydroxybenzaldehyde could regulate gut microbiota and inhibit lipid accumulation (Li et al., 2021). It can also sensitize *Acinetobacter baumannii* to antibiotics (Shin et al., 2018). The gut microbiota can metabolize tryptophan to produce indoles and their derivatives. They have the ability to upregulate the expression of genes critical for preserving the gut mucosal epithelial barrier and tight junctions in epithelial cells. Several bacteria participate in the degradation of these compounds, i.e., *Bacteroides,* etc. increased and *Akkermansia,* etc., decreased. These bacteria and metabolites and the process of their metabolism may contribute to maintaining homeostasis after PHx and promoting liver regeneration.

Some limitations remain in this study. Firstly, this study only involved samples from mice, whereas human samples would provide more meaningful information. We believe the animal study could provide references for further clinical studies. Secondly, the earlier time points between 6 and 36 h will be included in the future study. Thirdly, the results will be more reliable and solid if targeted metabolomics is used further to correctly quantify the differential metabolites. Finally, the impact of particular gut bacterial species and the metabolites they produce on liver regeneration, as well as the underlying mechanisms need to be further elucidated. Further study is ongoing to try to answer these questions.

## 5 Conclusion

In conclusion, our study demonstrated that the gut microbiota composition and gut microbiota-derived metabolites in the gut content changed markedly during liver regeneration. Untargeted metabolomics showed that PHx significantly modulates the metabolites involved in glycolysis, the TCA cycle, arginine metabolism, glutathione metabolism, tryptophan metabolism, purine and pyrimidine metabolism, which were co-metabolized by the host and gut microbiota. In the host, steroid hormone biosynthesis is the most significantly changed pathway. For the gut microbiota, *Escherichia Shigella*, *Lactobacillus*, *Akkermansia*, and *Muribaculaceae* were changed considerably during liver regeneration. Aminobenzoate degradation pathway was the most significant gut microbiota-specific metabolic pathway during liver regeneration. To the best of our knowledge, this is the first thorough explanation of the metabolites derived from the gut microbiota and how they relate to changes in the gut microbiota during liver regeneration after 2/3 PHx. This discovery advances our knowledge of the metabolic mechanism underlying liver regeneration and offers a novel, potentially useful therapeutic target for it. Additional research will be done on the underlying role and mechanism of metabolites originating from gut bacteria in liver regeneration.

## Data Availability

The data presented in the study are deposited in the MetaboLights repository, accession number MTBLS10775.

## Supplementary material

SUPPLEMENTARY FIGURE S1 Typical LC-QTOF/MS chromatograms of gut content in positive **(A)** and negative modes.

SUPPLEMENTARY FIGURE S2 Metabolites (positive mode) in the gastrointestinal content significantly altered during liver regeneration following a 2/3 PHx. The volcano plots showed the Sham group compared with those of the 6 h group **(A)**, 36 h group **(B)**, 72 h group **(C)**, and 168 h group **(D)**.

SUPPLEMENTARY FIGURE S3 Metabolites (negative mode) in the gastrointestinal content significantly altered during liver regeneration following a 2/3 PHx. The volcano plots showed the Sham group compared with those of the 6 h group **(A)**, 36 h group **(B)**, 72 h group **(C)**, and 168 h group **(D)**.

SUPPLEMENTARY FIGURE S4 The heat map shows the significantly changed metabolites in the gut content in positive mode.

SUPPLEMENTARY FIGURE S5 The heat map shows the significantly changed metabolites in the gut content in negative mode.

SUPPLEMENTARY FIGURE S6 The box plot shows the typical significantly changed metabolites in the gut content.

SUPPLEMENTARY FIGURE S7 The parameters of regularized Canonical Correlation Analysis (rCCA) between gut microbiota and its metabolites.
