# Supplementary figures and images for: The integrated analysis of gut microbiota and metabolome revealed steroid hormone biosynthesis is a critical pathway in liver regeneration after 2/3 partial hepatectomy
Source: Front Pharmacol. 2024 Aug 12;15:1407401. doi: 10.3389/fphar.2024.1407401 (PMC11345278; doi:10.3389/fphar.2024.1407401)

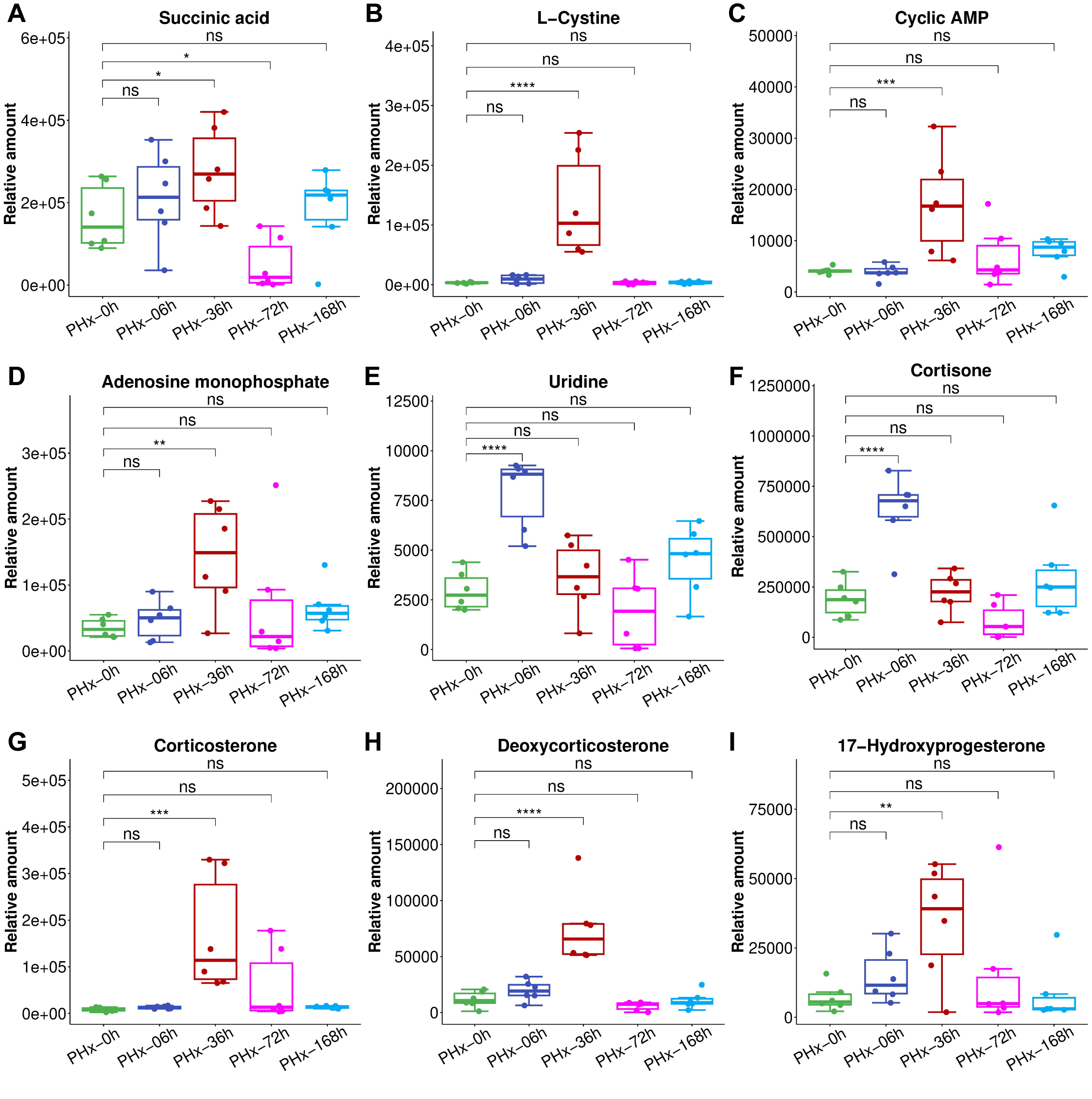

Supplement: Supplementary file 1 [file Image6.TIF]

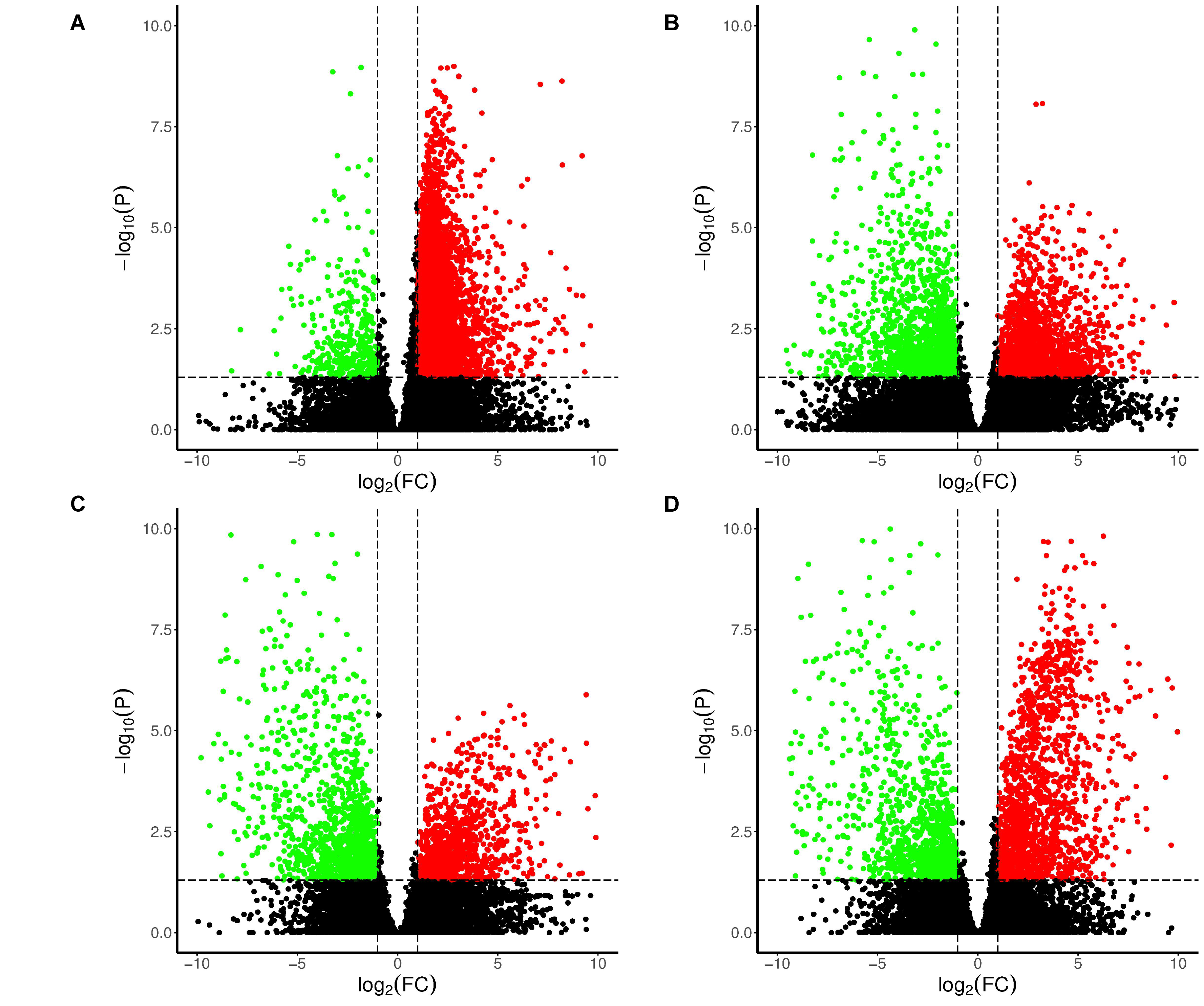

Supplement: Supplementary file 2 [file Image3.TIF]

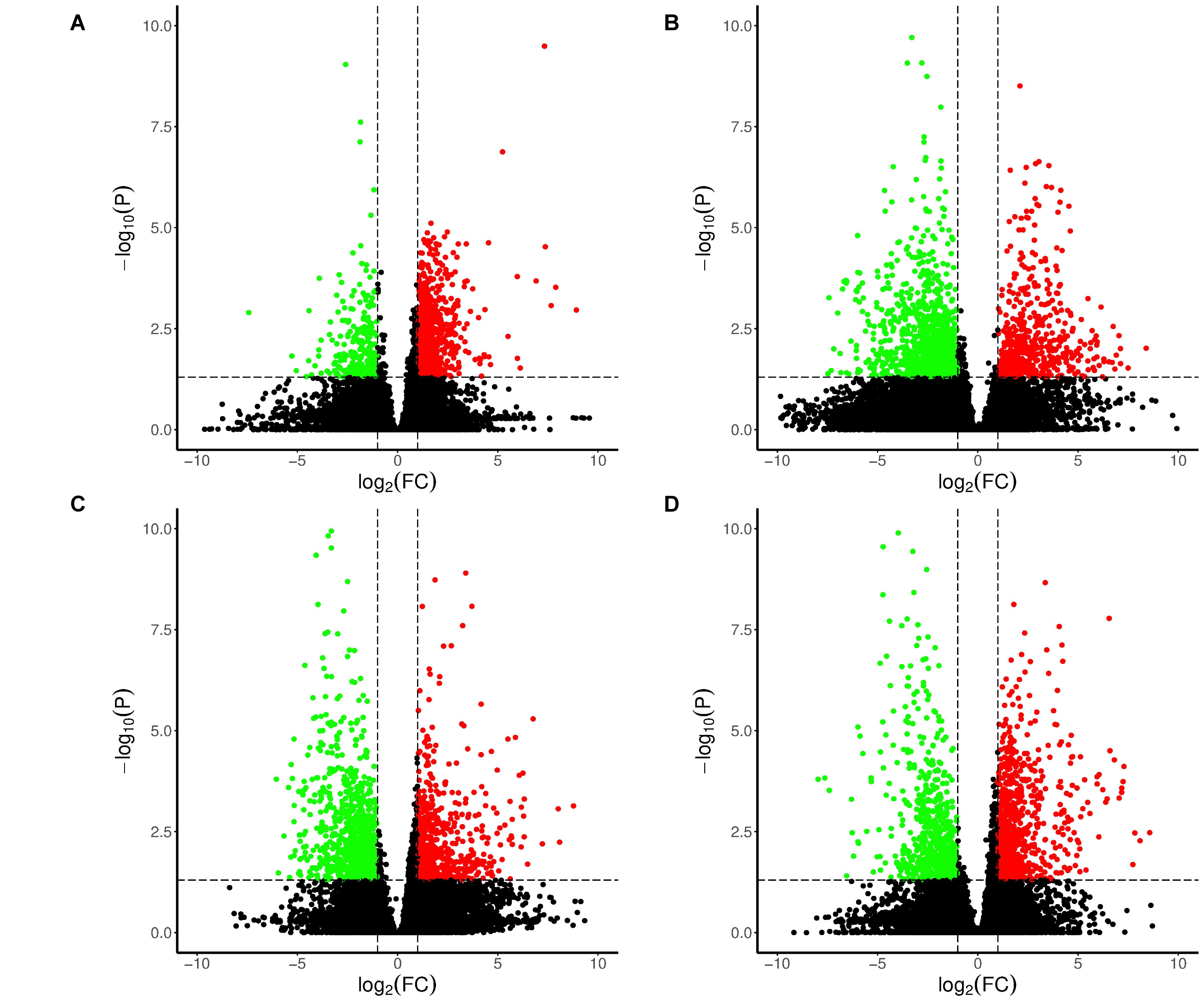

Supplement: Supplementary file 4 [file Image2.TIF]

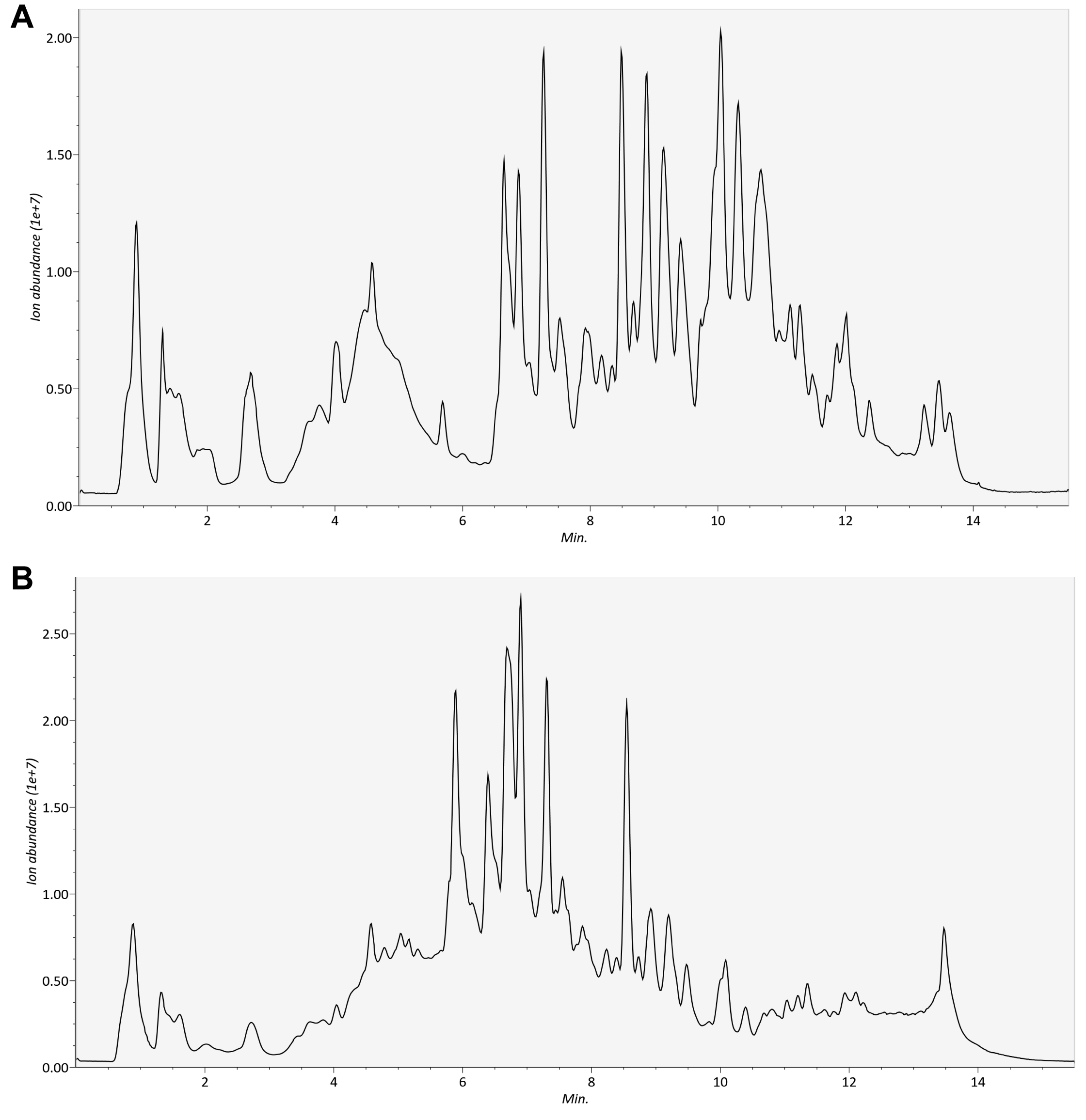

Supplement: Supplementary file 5 [file Image1.TIF]

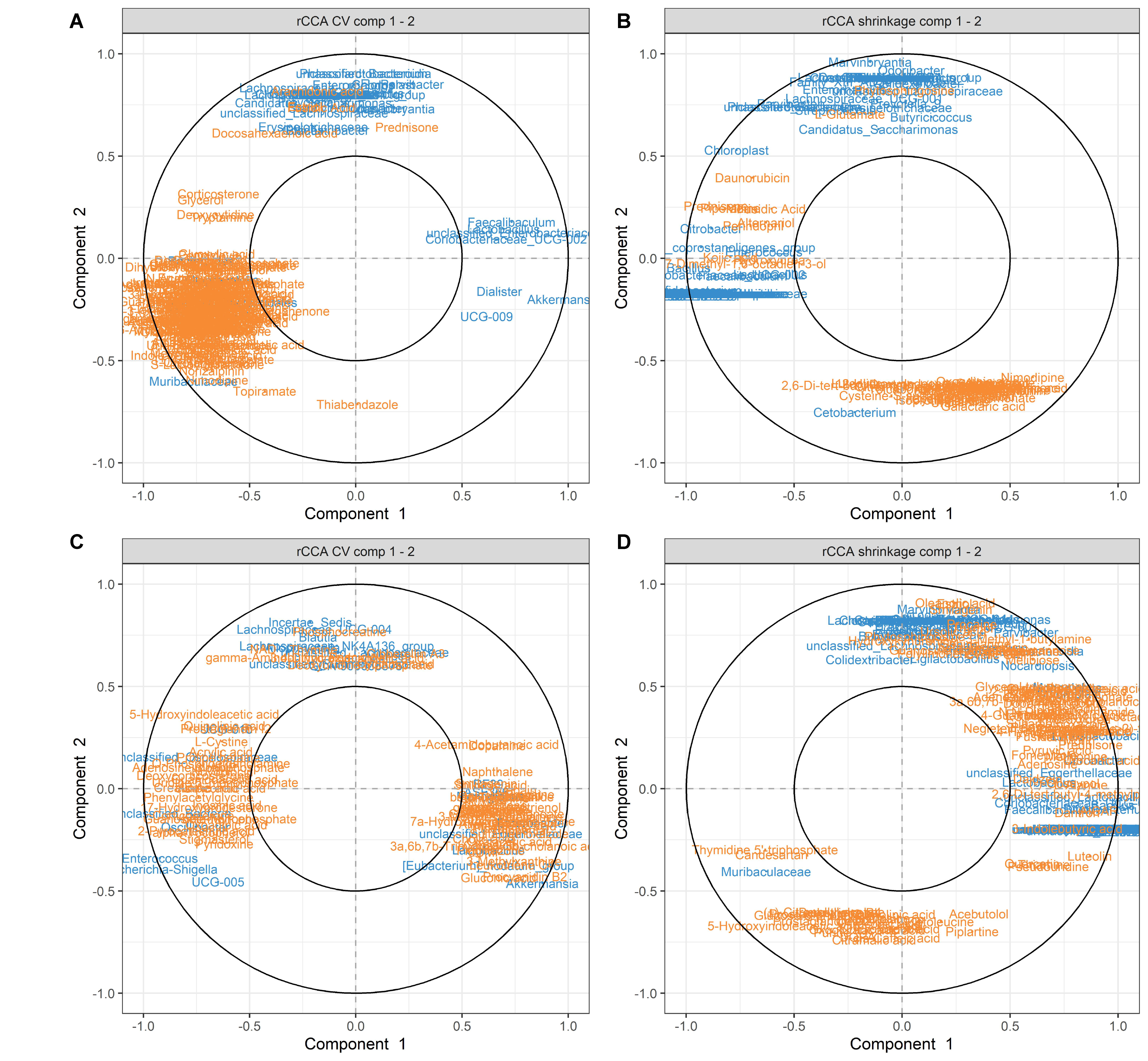

Supplement: Supplementary file 6 [file Image7.TIF]
