# Supplementary material for: The integrated analysis of gut microbiota and metabolome revealed steroid hormone biosynthesis is a critical pathway in liver regeneration after 2/3 partial hepatectomy
Source: Front Pharmacol. 2024 Aug 12;15:1407401. doi: 10.3389/fphar.2024.1407401 (PMC11345278; doi:10.3389/fphar.2024.1407401)
Supplement: Supplementary file 8 [file DataSheet1.docx]

**The Integrated Analysis of Gut Microbiota and Metabolome Revealed Steroid Hormone Biosynthesis is a Critical Pathway in Liver Regeneration after 2/3 Partial Hepatectomy**

Runbin Sun^1, 2 *^, Fei Fei^1, 2^, Dandan Jin^2^, Haoyi Yang^2^, Zhi Xu^1, 2^, Bei Cao^1, 2†^, Juan Li^2*^

1. Phase I Clinical Trials Unit, Nanjing Drum Tower Hospital, Affiliated Hospital of Medical School, Nanjing University, Nanjing, China, 210008
2. Phase I Clinical Trials Unit, Nanjing Drum Tower Hospital, Nanjing Drum Tower Hospital Clinical College of Nanjing University of Chinese Medicine, Nanjing, China, 210023

* Correspondence:

Runbin Sun, Email: runbinsun@gmail.com; Tel.: 025-83106666,

Phase I Clinical Trials Unit, Nanjing Drum Tower Hospital, Affiliated Hospital of Medical School, Nanjing University, Nanjing, China, 210008

Juan Li, Email: juanli2003@njglyy.com; Tel.: 025-69588516,

Phase I Clinical Trials Unit, Nanjing Drum Tower Hospital, the Affiliated Hospital of Nanjing University Medical School, Nanjing, China, 210008

**Supplementary methods**

**Materials**

4-chlorophenylalanine was purchased from Aladdin Biochemical Technology Co., Ltd. (Shanghai, China). Methanol and acetonitrile were purchased from Merck KGaA (Darmstadt, Germany).

**Animals and Treatment**

Thirty mice were divided into 5 groups using the random number table method, with 6 mice in each group. After anesthesia with isoflurane, the mouse abdomen was skinned and disinfected, and the skin and muscles were incised along the midline of the abdomen under the xiphoid process to enter the abdominal cavity with a length of 2 cm-3 cm. Avoid damaging the abdominal organs and tissues during the laparotomy. The homemade retractor exposes the surgical field, fully exposes the liver, and frees the liver with gentle movements. Mice in the liver resection group were ligated with 5-0 silk thread at the roots of the left and middle lobes of the liver respectively. The ligation should be close to the base of the liver lobes to minimize the residual liver lobes and damage to blood vessels. The movements should be gentle during ligation to avoid tearing. After the liver lobes are successfully ligated, the color of the liver lobes will darken within a few seconds, and then both lobes are removed. The abdominal cavity is flushed with sterile saline to reduce contamination. After cleaning the abdominal cavity, the abdomen is closed in two layers and disinfected again after closing. Make the incision, remove the anesthesia machine, and wait for the mouse to wake up. Because the mice are small in size, their tissues are fragile, and the liver tissue is easily damaged during the operation, so gentle movements are required. In addition, in order to prevent the occurrence of postoperative hypothermia, mice must be placed on a warming pad to maintain a warmer constant temperature environment. The control group (sham operation group) only underwent laparotomy and suturing. The clinical manifestations were observed after the operation. The patients were weighed and sacrificed 6 h, 36 h, 72 h, and 168 h after liver resection. The livers were weighed and the liver index was calculated. The anticoagulated blood was centrifuged at 8000 rpm for 5 minutes and followed by reagents. The whole blood was centrifuged at 8000 rpm for 5 min to get the serum and was stored at -80℃ for further analysis. Livers were harvested and weighed, the liver index was calculated (liver weight/body weight). Levels of serum total bile acids (TBA), alanine aminotransferase (ALT), aspartate aminotransferase (AST) and alkaline phosphatase (ALP) were measured.

**The parameters of LC-QTOF/MS analysis**

The column used is ACQUITY UPLC@HSS T3 HPLC column (1.8 µm; 2.1 mm × 100 mm; Waters, USA). The temperature of the column oven was set to 40°C. The LC-Q/TOF-MS consisted of an LC system (ExionLC, Foster City, CA) coupled with a hybrid quadrupole time-of-flight tandem mass spectrometer (AB SCIEX TripleTOF® 5600 LC-Q/TOF-MS, Foster City, CA). The mobile phase consisted of solvent A (water with 0.1% formic acid) and solvent B (acetonitrile with 0.1% formic acid) with the following gradient: 0-1.5 min 5% B, 1.5-2.5 min 5-15% B, 2.5-6 min 15-60% B, 6-10 min 60-95% B, 10-12 min 95% B, 12-12.5 min 95-5% B, 12.5-15.5 min 5% B. The flow rate was 0.4 mL/min. The mass detection was performed in positive (4500 V) and negative (-5500 V) ion modes for scan analysis with Turbo V electrospray ionization (ESI). The parameters were set as follows: ion spray voltage, 7 kV; turbo spray temperature (TEM), 550℃; declustering potential (DP), 70 V; collision energy (CE), 35 eV; nebulizer gas (gas1), 55 psi; heater gas (gas 2), 55 psi; curtain gas, 35. The nebulizer and auxiliary gas were kept by Nitrogen. TOF MS ranged from m/z 50-1200. Automatic calibration was carried out every twenty samples.

**Compound identification**

MS-DIAL 4.90 (http://prime.psc.riken.jp) was used for peak detection, spectral deconvolution, peak alignment and compound identification. Databases including HMDB (https://hmdb.ca/), METLIN (http://metlin.scripps.edu), and an in-house database established in our lab were used to identify and interpret chromatographic peaks. The metabolites were identified by comparing the detected mass spectra and retention time of the compounds in the database.

**Multivariate and univariate data analysis**

Peak areas of detected compounds were normalized by the area of IS for each sample. Principal components analysis (PCA) and partial least squares discriminant analysis (PLS-DA) were performed using the mixOmics package of the R project (version 4.2.1). The PCA model was used to see the overall distribution of all the samples, and the PLS-DA model was used to confirm the general separation among the five groups. Variable of importance in the project (VIP) analysis was used to identify the endogenous metabolites contributing to the classification. Furthermore, univariate analysis was conducted by one-way analysis of variance (ANOVA) followed by a pairwise t-test and corrected by the Benjamini-Hochberg method to control the False Discovery Rate (FDR). Peaks that had a p-value less than 0.05 were considered to be differential. The differential metabolites selected by multivariate and univariate data analysis were further analyzed by fold change among the four groups.

**16S rDNA gene sequencing**

Genomic DNA from the gut content of mice in each group was extracted following the protocol of Fast DNA™ SPIN Kit (MP Biomedicals, CA, USA). Briefly, about 100 mg of mouse gut content sample was weighed and the total DNA was extracted and quantified by Nanodrop. The target fragment was amplificated by high-fidelity PCR. Then the amplified products were purified and recovered with magnetic beads. The recovered products of PCR amplification were quantified by fluorescence with the fluorescent reagent Quant-iT PicoGreen dsDNA Assay Kit, and the quantitative instrument was a Microplate reader (BioTek, FLx800). According to the quantitative fluorescence results, the samples were mixed according to the corresponding proportion according to the sequencing volume requirement of each sample. The sequencing library was prepared with TruSeq Nano DNA LT Library Prep Kit (Illumina, San Diego, CA, USA). The quality inspection of the sequencing library was performed on the Agilent Bioanalyzer using the Agilent High Sensitivity DNA Kit. The library was quantified using the Quant-iT PicoGreen dsDNA Assay Kit on the Promega QuantiFluor fluorescence quantification system.
